# Does the Use of Intravenous Immunoglobulin Improve Clinical Outcomes in Adults With Autoimmune Encephalitis? A Systematic Review

**DOI:** 10.1002/brb3.70491

**Published:** 2025-05-12

**Authors:** Anahat Kalra, Olivia Mackay, Emma Thomas‐Jones, Tom Solomon, Paula Foscarini‐Craggs

**Affiliations:** ^1^ School of Medicine Cardiff University Cardiff UK

**Keywords:** autoimmune encephalitis, intravenous immunoglobulin

## Abstract

**Background:**

Autoimmune encephalitis is an immune‐mediated inflammatory condition affecting the central nervous system. Current best‐practice guidelines recommend intravenous immunoglobulin (IVIG) for use if corticosteroids are ineffective, but the evidence surrounding the efficacy of IVIG in autoimmune encephalitis is yet to be evaluated.

**Objectives:**

Perform a systematic review of the available literature to evaluate the efficacy of IVIG in autoimmune encephalitis and the impact of IVIG on clinical outcomes.

**Methods:**

This systematic review was written following PRISMA‐S guidelines. Papers eligible for inclusion were primary research including adults aged 16 years and above, with a suspected or confirmed diagnosis of autoimmune encephalitis, treated using IVIG. Each included study was assessed for quality.

**Results and conclusion:**

From 2533 records, 27 studies met the inclusion criteria. The use of IVIG is associated with improved short‐term and long‐term neurological outcomes. There is evidence that IVIG may be more effective when used in combination with corticosteroids. The use of IVIG is significantly associated with reduced seizure incidence in patients. Where recorded, adverse effects related to IVIG were few. There is insufficient data available on how IVIG impacts mortality rates, discharge rates, and its effects according to different types of autoimmune encephalitis. Out of the included papers, there was 1 randomized controlled trial (RCT), 3 nonrandomized trials and 23 cohort studies. 25 of 27 papers were deemed to have a high or serious risk of bias. Heterogeneous study design and the quality of studies were major limiting factors for this review, highlighting the need for further research.

## Graphical abstract

Evidence surrounding the use of IVIG in treating autoimmune encephalitis has yet to be evaluated and yet IVIG is used second line in this condition, after corticosteroids. IVIG is prone to donor shortages and therefore it would prove beneficial to establish whether this is an effective treatment; it can then be prioritised for this condition or others.

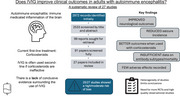

**Abbreviations**
ACE: Addenbrooke's Cognitive Exam
AE: Autoimmune encephalitis
CI: Confidence Interval
DVT: Deep venous thrombosis
EEG: Electroencephalogram
IVIG: Intravenous Immunoglobulin
LGI‐1: Leucine‐rich, glioma‐inactivated 1
MMSE: Mini‐Mental State Examination
MoCA: Montreal Cognitive Assessment
MRI: Magnetic resonance imaging
MRS: Modified Rankin Score
NMDAR: N‐Methyl‐D‐aspartate receptor
NOS: Newcastle–Ottawa scale
PRISMA: Preferred Reporting Items for Systematic Reviews and Meta‐Analysis
RCT: Randomized controlled trial
ROB: Risk of bias

## 1 Introduction

Autoimmune encephalitis is the name given to a group of conditions involving immune‐mediated inflammation of the brain parenchyma. The encephalitis can be associated with various anti‐neuronal antibodies which are responsible for attacking different structures within the central nervous system (Vincent 2009), including those against the N‐Methyl‐D‐aspartame (NMDAR) receptor.

**FIGURE 1:**
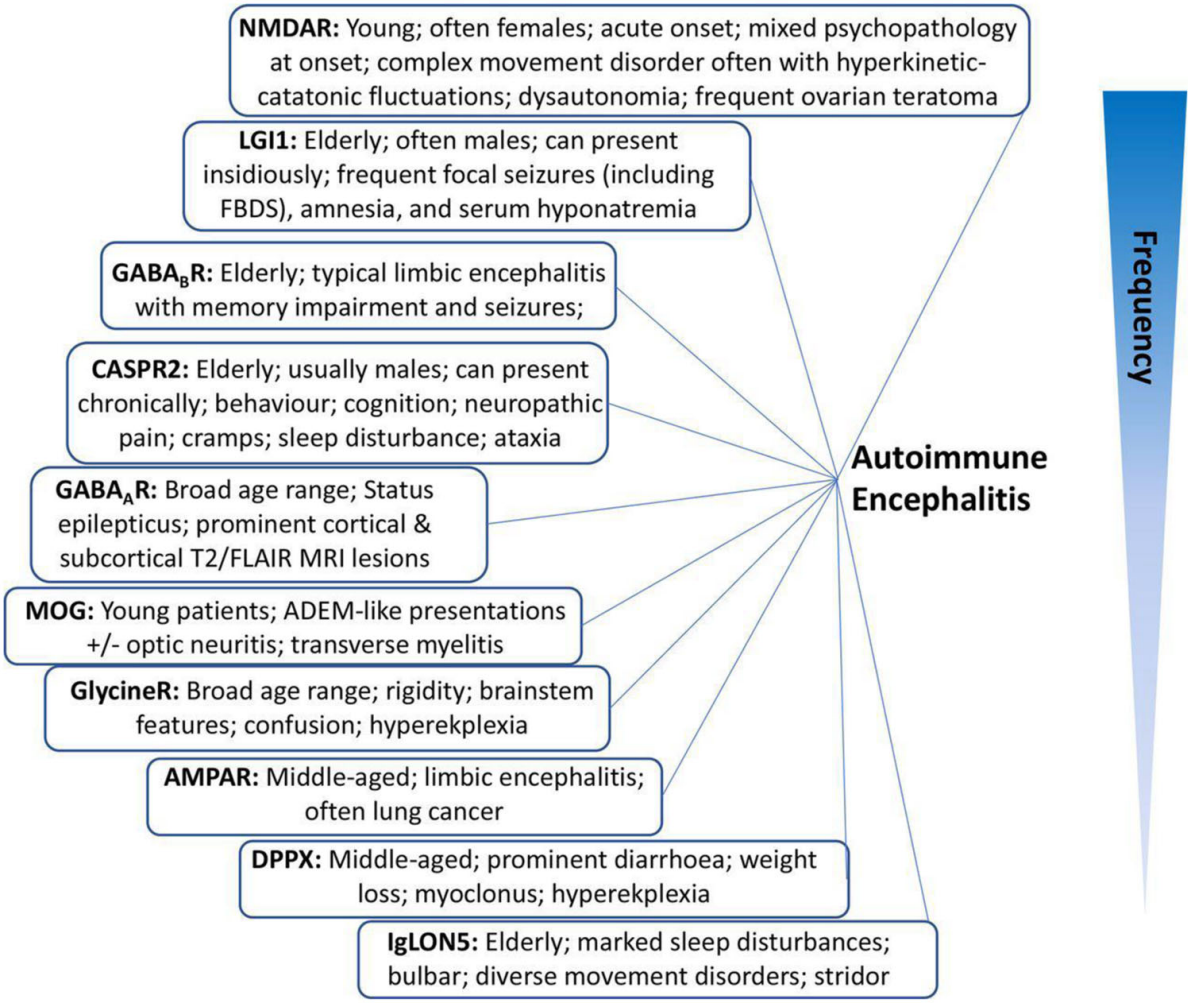
Classic syndromes and characteristic features of neuronal autoantibodies (Uy et al. 2021).

Due to the heterogeneity of these conditions, it can be difficult to identify who is at risk of developing this pathology. Researchers at Oxford University have compiled this graphic (Figure 1) “identifying” clinical features (Uy et al. 2021) which may prove useful to clinicians when presented with a case that could be autoimmune encephalitis. However, due to the multiple possible presentations, it is important for clinicians to consider the differential of autoimmune encephalitis when presented with any patient with neuro‐psychiatric symptoms.

It is not a common group of conditions, with around 1000 cases per year being diagnosed in the United Kingdom (Ellul et al. 2020), but the consequences, including seizures, cognitive impairment and psychiatric symptoms (Abboud et al. 2022) require long‐term management. Current best practice guidelines indicate the use of corticosteroids first due to their broad immunosuppressive effect (Hesham et al. 2021). However, additional treatments including intravenous immunoglobulin (IVIG) and plasma exchange are usually given as an adjunct (Hesham et al. 2021), though there are few randomized controlled data on any of these treatments. Determining which treatments are effective could have a significant impact on the lives of people affected by autoimmune encephalitis.

IVIG has been proven to be effective in treating other autoimmune conditions of the peripheral nervous system, such as myasthenia gravis and Guillain–Barré syndrome, and is used first line in the latter condition (Leonhard et al. 2019). There are many possible mechanisms of action of IVIG, but it has been suggested that IVIG neutralizes or downregulates pathogenic autoantibodies, which would suggest it to be an effective therapy in autoimmune encephalitis.

Because IVIG is a blood product, there are limitations in its use. Blood products are dependent on donors and therefore prone to shortages. Due to this, in the United Kingdom they are rationed for specific diseases by a commissioning group. Blood products are also expensive and can have significant adverse effects such as thromboembolic events, which have been suggested to be associated with the use of IVIG (Mahima et al. 2022). Currently, IVIG is permitted for use in autoimmune encephalitis if treatment with corticosteroids is not effective (NHS England Immunoglobulin Expert Working Group 2021). However, a search of the current literature indicates there is a lack of robust evidence synthesis surrounding the topic of IVIG being used in this condition, making it difficult for clinicians to find evidence‐based guidance on this intervention.

Clinicians and governing bodies would benefit greatly from IVIG being ruled as effective or ineffective in treating this disease, as if proven effective, it would allow its use to be prioritized in this disease while the contrary would allow it to be prioritized for other conditions and would avoid use of an unnecessary, expensive, and potentially dangerous treatment.

A review of the literature is required to see what the current evidence states about the use of IVIG in autoimmune encephalitis. Thorough appraisal of the current literature can pave the way for future randomized control trials as well as national and international clinical guidelines, reducing uncertainty for both clinicians and patients. This systematic review will evaluate the current evidence available to understand whether IVIG improves clinical outcomes in adults with autoimmune encephalitis, to answer the following questions:

■ How does the use of IVIG affect short‐term and long‐term neurological outcomes?
■ Which adverse events are associated with the use of IVIG?
■ How does the use of IVIG affect the incidence of seizures?
■ How does the use of IVIG affect rates of mortality and discharge?
■ How does the use of IVIG impact the use of additional treatments compared with using corticosteroids alone?
■ Do different antibody types in autoimmune encephalitis affect clinical outcomes when using IVIG?

## 2 Methodology

This systematic review was conducted and reported according to PRISMA guidelines. A protocol was prepared before commencing the review and prospectively registered with the PROSPERO database (ID: CRD42023395827)

### 2.1 Searches

Electronic searches were conducted in Ovid MEDLINE, Embase, Web of Science, and The Cochrane Library. The key words used included “autoimmune encephalitis,” and “encephalitis,” combined with “IVIG,” and “Intravenous immunoglobulins.” The full search strategy can be found in the protocol. The publication dates were limited between January 1970 to December 2022 and the published language was limited to English. This beginning date was chosen as immunoglobulins were not widely therapeutically used before this.

The decision was made to use the word “encephalitis” as a key word as although this opened up the results to far more etiologies than autoimmune, there was a risk of missing papers that may refer to the disease specifically by antibody type, for example, anti LGI1 encephalitis, rather than autoimmune encephalitis. By opening up the search, the risk of losing these studies was mitigated.

### 2.2 Study Selection

Articles retrieved from electronic searches were screened for eligibility using their titles and abstracts by the review author (AK). 20% of titles and abstracts were independently screened by a second reviewer (OM).

Subsequently included full papers were screened using the same criteria, with 20% of full papers being screened independently by the second reviewer.

Discrepancies in this process were identified after each stage and resolved by discussion between the two reviewers.

The inclusion criteria were as follows:

■ *Population*: Adult patients (16 years and older) with clinical or laboratory diagnosis of autoimmune encephalitis, globally.
■ *Intervention*: Some or all participants were treated with intravenous immunoglobulin.
■ *Comparator*: None specified.
■ *Outcomes*: Any clinical outcome concerning treatment.

Some specific exclusions included:

■ Studies that solely discussed children—although studies with a mixed population of adults and children were still considered and included if they provided relevant clinical outcomes and specified the treatment of the adult patients.
■ Encephalitis etiology that is not immune, for example, viral encephalitis or wider conditions such as encephalomyelitis.

Study designs included: randomized controlled trials (RCT), nonrandomized clinical trials, retrospective and prospective cohort studies, cross‐sectional studies, case‐control studies.

Study designs excluded: nonprimary research (reviews, opinion pieces, editorials), animal studies, case series, case reports, grey literature, and conference abstracts where there was no full paper available.

### 2.3 Data Extraction

Relevant data from included studies were extracted and tabularized (see Supplementary Material). Extracted data included the following details: citation, study type, study objective, country, participant number, participant demographics, randomization (Y/N), blinding (Y/N), the subtypes of autoimmune encephalitis included, immunotherapies used, how many participants had IVIG, additional treatments and therapies, time to discharge, mortality rates, duration of follow‐up, outcome measures, results reported, statistical methods used, key limitations, study funding sources, and conflicts of interest for the authors.

### 2.4 Assessment of Quality

Included studies were classified based on study design. The methodological quality of the observational studies was assessed using the Newcastle–Ottawa quality assessment scale (NOS) (Wells et al. 2000), for which each study was given a score from 0–9. A study with a score from 7–9 was deemed high quality, 4–6 was deemed high risk of bias, and 0–3 was deemed very high risk of bias.

The Cochrane Risk of Bias (ROB2) tool (Sterne et al. 2019) and the Cochrane Risk Of Bias In Non‐randomized Studies of Interventions (ROBINS‐I) tool (Sterne et al. 2016) were used to assess RCTs and nonrandomized trials respectively.

### 2.5 Synthesis and Analysis of Results

Due to the anticipated heterogeneity between study designs and the reporting of outcome measures, a narrative synthesis was used to analyze the research. An exploratory approach was used, considering factors such as study design, sample size, and outcomes measured in the synthesis. Where multiple studies considered the same outcomes, studies of higher quality were discussed in more detail.

## 3 Results

### 3.1 Search Results and Eligible Studies

**FIGURE 2:**
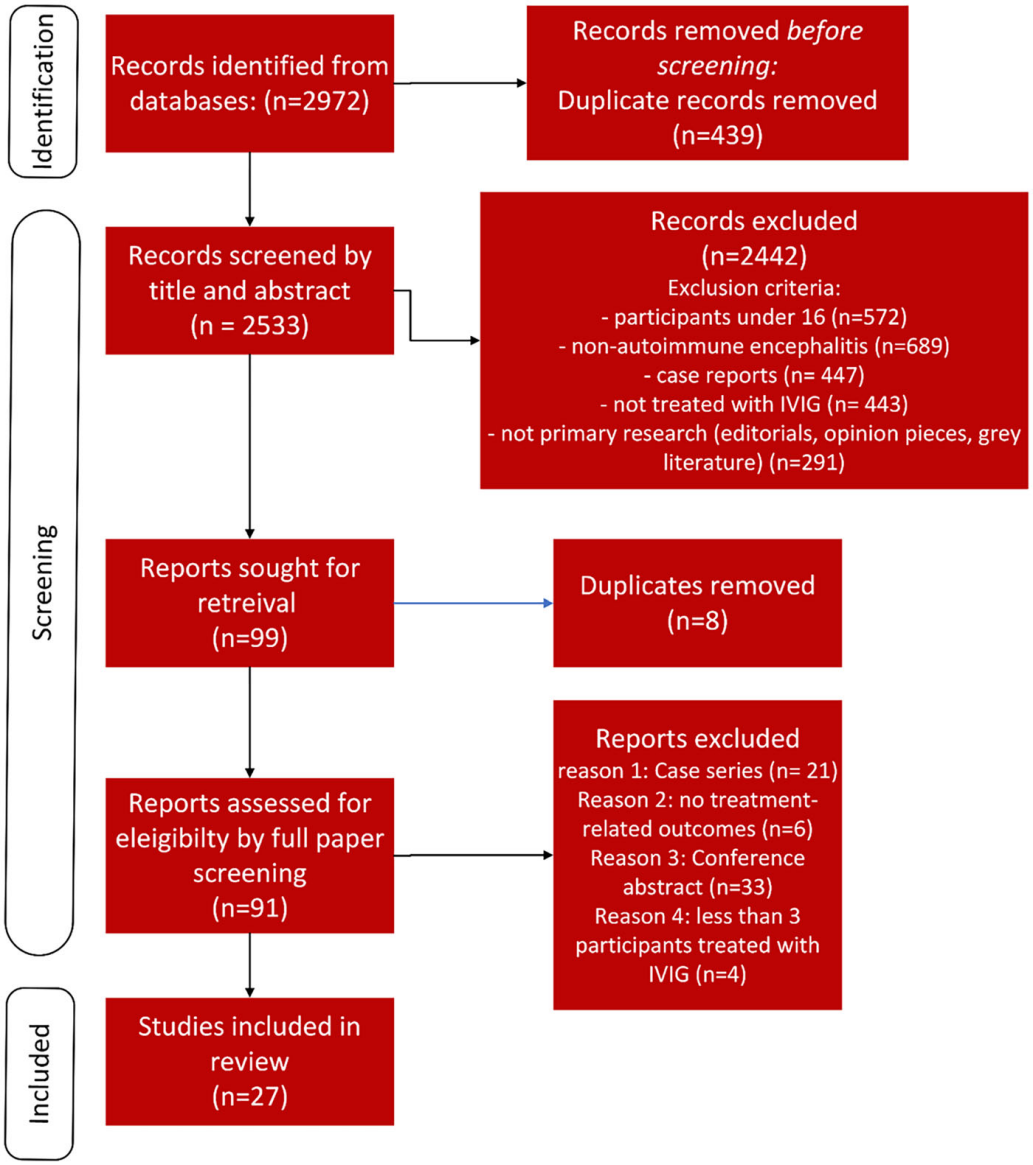
Flowchart of study selection following the Preferred Reporting Items for Systematic Reviews and Meta‐Analysis (PRISMA) protocol.

Using the search strategy and parameters, a total of 2533 unique records were identified after de‐duplication. Of these, 27 were included in the final review. A detailed description of excluded studies is shown in Figure 2.

### 3.2 Study Characteristics

In the final analysis, there was 1 randomized controlled trial (Dubey et al. 2020), 3 nonrandomized trials (Lee et al. 2022; Wong et al. 2010; Zhang et al. 2019) and 23 cohort studies—8 prospective (Dai et al. 2019; Gong et al. 2022; Huang et al. 2016; Huang et al. 2022; Lee et al. 2021; Pradhan et al. 2019; Titulaer et al. 2013; Zhang et al. 2018) and 15 retrospective (Ariño et al. 2016; Cai et al. 2022; Hang et al. 2020; Huang et al. 2015; Kong et al. 2019; Lee et al. 2022; Melamud et al. 2020; Pham et al. 2011; Shin et al. 2013; Wang et al. 2021; Wang et al. 2021; Xu et al. 2021; Yoshikawa et al. 2020; Zhu et al. 2020; Zhu et al. 2020). A total of 2236 patients were described, with a range of 7–501 patients and a median of 31 patients per study.

For a summary of studies, see **Table** **1**.

**TABLE 1:** Summary of included studies (Sterne et al. 2016; Dubey et al. 2020; Lee et al. 2022; Wong et al. 2010; Zhang et al. 2019; Dai et al. 2019; Gong et al. 2022; Huang et al. 2016; Huang et al. 2022; Lee et al. 2021; Pradhan et al. 2019; Titulaer et al. 2013; Zhang et al. 2018; Ariño et al. 2016; Cai et al. 2022; Hang et al. 2020; Huang et al. 2015; Kong et al. 2019; Lee et al. 2022; Melamud et al. 2020; Pham et al. 2011; Shin et al. 2013; Wang et al. 2021; Wang et al. 2021; Xu et al. 2021; Yoshikawa et al. 2020; Zhu et al. 2020) collated from extracted data (see Supplementary Material).

Author (year)	Study type	Sample size	Outcome measure
Arino et al. (2016)	Retrospective cohort	76	Cognitive performance score
Cai et al. (2022)	Retrospective cohort	78	mRS
Dai et al. (2019)	Prospective cohort	108	mRS
Dubey et al. (2020)	RCT	17	Seizure frequency + RBANS
Gong et al. (2022)	Prospective cohort	347	mRS
Hang et al. (2020)	Retrospective cohort	21	MMSE and MoCA‐B scores
Huang et al. (2016)	Prospective cohort	29	Psychiatric/neurological status
Huang et al. (2015)	Retrospective cohort	33	mRS
Huang et al. (2022)	Prospective cohort	43	mRS
Kong et al. (2019)	Retrospective cohort	24	mRS
Lee et al. (2022)	Single‐arm trial	18	mRS
Lee et al. (2021)	Prospective cohort	78	CASE and mRS
Lee et al. (2022)	Retrospective cohort	147	CASE and mRS
Melamud et al. (2020)	Retrospective cohort	10	mRS
Pham et al. (2011)	Retrospective cohort	9	Neurological status
Pradhan et al. (2019)	Prospective cohort	31	Symptom resolution
Shin et al. (2013)	Retrospective cohort	14	mRS
Titulaer et al. (2013)	Prospective cohort	577	mRS
Wang et al. (2021)	Retrospective cohort	278	In‐hospital infection rate
Wang et al. (2021)	Retrospective cohort	56	seizure frequency, mRS
Wong et al. (2010)	Single‐arm trial	9	Antibody titers, MRI brain
Xu et al. (2021)	Retrospective cohort	31	mRS
Yoshikawa et al. (2020)	Retrospective cohort	83	Functional Grade
Zhang et al. (2018)	Prospective cohort	111	mRS
Zhang et al. (2019)	Nonrandomized trial	40	mRS
Zhu et al. (2020)	Retrospective cohort	7	No measure specified
Zhu et al. (2020)	Retrospective cohort	14	No measure specified

### 3.3 Risk of Bias

As the study type varied, the Newcastle–Ottawa scale (NOS) (Wells et al. 2000) the Cochrane Risk of bias (ROB2) tool, (Sterne et al. 2019), and the Risk of Bias in Non‐Randomized Studies of Interventions (ROBINS‐I) (Sterne et al. 2016) tool were used for each study design.

Apart from one cohort study, the rest of the observational studies were deemed either high risk or very high risk of bias. In most cohort studies, the risk of bias was high due to a lack of detail regarding the derivation of the exposed and unexposed cohorts, and a lack of comparability between groups of patients.

The cohort study by Gong et al. (2022) is a prospective cohort with propensity score matching carried out before analysis to create triplets of participants with matching baseline characteristics. This resulted in comparable patient groups. The study also consisted of a control group with no IVIG used as treatment as part of the comparison. For these reasons, this study achieved a high‐quality rating.

Out of the included trials, the randomized trial (Dubey et al. 2020) had a low risk of bias due to the randomization, allocation concealment, and blinding. The other three trials, which had no randomization or blinding, were deemed to be of a moderate to serious risk of bias.

The risk of bias assessments for each study is summarized in Tables 2 and 3.

**TABLE 2:** Risk of bias assessment for included observational studies (Zhang et al. 2019; Dai et al. 2019; Gong et al. 2022; Huang et al. 2016; Huang et al. 2022; Lee et al. 2021; Pradhan et al. 2019; Titulaer et al. 2013; Zhang et al. 2018; Ariño et al. 2016; Cai et al. 2022; Hang et al. 2020; Huang et al. 2015; Kong et al. 2019; Lee et al. 2022; Melamud et al. 2020; Pham et al. 2011; Shin et al. 2013; Wang et al. 2021; Wang et al. 2021; Xu et al. 2021; Yoshikawa et al. 2020; Zhu et al. 2020), assessed using the Newcastle–Ottawa Tool (NHS England Immunoglobulin Expert Working Group 2021).

Study	Selection (max 4)	Comparability (max 2)	Outcome (max 3)	Total out of 9	Risk of bias
Arino et al. (2016)	3	0	2	5	High risk
Cai et al. (2022)	3	0	3	6	High risk
Dai et al. (2019)	3	0	3	6	High risk
Gong et al. (2022)	4	1	3	8	High quality
Hang et al. (2020)	3	0	3	6	High risk
Huang et al. (2016)	2	0	3	5	High risk
Huang et al. (2021)	3	0	3	6	High risk
Huang et al. (2015)	2	0	2	4	High risk
Kong et al. (2019)	2	0	3	5	High risk
Lee et al. (2021)	3	0	3	6	High risk
Lee et al. (2022)	3	0	3	6	High risk
Melamud et al. (2020)	3	0	3	6	High risk
Pham et al. (2011)	3	0	2	5	High risk
Pradhan et al. (2019)	3	0	3	6	High risk
Shin et al. (2013)	3	0	2	5	High risk
Titulaer et al. (2013)	2	0	3	5	High risk
Wang et al. (2021)	3	0	2	5	High risk
Wang et al. (2021)	2	0	2	4	High risk
Xu et al. (2021)	3	0	3	6	High risk
Yoshikawa et al. (2020)	3	0	1	4	High risk
Zhang et al. (2017)	3	0	3	6	High risk
Zhu et al. (2020)	2	0	0	2	Very high risk
Zhu et al. (2020)	3	0	0	3	Very high risk

**TABLE 3:** Risk of bias assessment for included trials (Sterne et al. 2016; Dubey et al. 2020; Lee et al. 2022; Wong et al. 2010), assessed using the ROB2 (Wells et al. 2000) tool and the ROBINS‐I (Sterne et al. 2019) tool.

Study	Study type	Risk of bias tool	Overall risk of bias
Dubey et al. (2020)	RCT	ROB2	Low
Lee et al. (2022)	Single arm open label trial	ROBINS‐I	Moderate/serious
Zhang et al. (2019)	Two‐arm open label trial	ROBINS‐I	Moderate/serious
Wong et al. (2010)	Single arm open label trial	ROBINS‐I	Moderate/serious

The results of the papers have been synthesized to answer the following research questions:

o How does the use of IVIG affect neurological outcomes: short‐term and long‐term?
o Which adverse events are associated with the use of IVIG?
o How does the use of IVIG affect the incidence of seizures?
o How does the use of IVIG impact the use of additional treatments compared with using corticosteroids alone?
o How does the use of IVIG affect time to discharge and mortality rates?
o Do different antibody types in autoimmune encephalitis affect clinical outcomes when using IVIG?

### 3.4 Neurological and Cognitive Outcomes

Symptoms of autoimmune encephalitis are primarily neurological or cognitive in nature. Fifteen out of 27 studies used the Modified Rankin Scale (mRS) to assess neurological outcome measures. The mRS is a six‐point scale used to assess the degree of disability or dependence in daily activities of individuals who have suffered a stroke or a neurological disability. In all included studies, a score of 0–2 was considered a good outcome and a score of 3–6 was considered a poor outcome. Other scoring systems used to evaluate neurological and cognitive outcomes were the Addenbrooke's cognitive exam (ACE), the Montreal Cognitive Assessment B (MoCA‐B), the mini‐mental state exam (MMSE), the repeatable battery for assessment of neuropsychological status (RBANS), the cognitive performance scale (CPS) as well as simpler in‐house measurements based on patients’ neurological symptoms improving or resolving.

Neurological assessments can be split into short‐term and long‐term outcomes. For this review, less than 6 months was considered “short‐term” and 6 months or greater was considered “long‐term.”

#### 3.4.1 Short‐Term Outcomes

The randomized controlled trial (Dubey et al. 2020) reported that at week 5, a larger proportion of patients—8/8—treated with IVIG achieved stable/improved Repeated Battery for the Assessment of Neuropsychological Status (RBANS) scores compared to 5/8 patients in the placebo group. However, this difference was not statistically significant (*p* = 0.100).

The high‐quality cohort study (Gong et al. 2022) reported that after propensity score matching of baseline characteristics, the proportion of patients who achieved mRS score improvement greater than one point after one month was the highest, at 86.5%, in those treated with a combination of intravenous methylprednisolone and IVIG compared to those treated with monotherapy of methylprednisolone or IVIG—55.6% and 68.7%, respectively. This difference was statistically significant (*p* < 0.01). Another cohort study of 78 participants similarly demonstrated that 3/11 (27.8%) treated with IVIG improved in terms of their mRS score and 3/11 (27.8%) treated with methylprednisolone improved at the 4‐week follow‐up while a much higher proportion of patients—20/56, (36%)—treated with both IVIG and methylprednisolone combination therapy improved.

One trial (Lee et al. 2022) demonstrated good outcomes within a week of IVIG treatment. 10/18 participants had a favorable mRS score on day 8 (*p* = 0.004). At day 29, 16/18 participants had a favorable mRS score (*p* = 0.0001), however, by this point, 12 participants had received rescue therapy due to either deteriorating mRS scores or unchanging mRS scores.

Four cohort studies (Huang et al. 2016; Zhang et al. 2018; Huang et al. 2015; Xu et al. 2021) did not separate treatment outcomes based on specified immunotherapies and instead summarized outcomes for the whole cohort. In all of these cohorts, over half of the participants were given IVIG as part of their treatment regime, often among other therapies. In all cohorts except one, the vast majority of patients, at percentages of 93.5%, 98.1%, and 77%, were reported to achieve favorable mRS scores in the short‐term within 1–3 months. In the remaining cohort study (Huang et al. 2016), 27.6% of patients reached the improvement grade of only mild symptoms at the one‐month follow‐up, however, this study also included children.

#### 3.4.2 Long‐Term Outcomes

Gong et al. (2022) showed significantly lower mRS scores at 12 months follow‐up in the combined IVIG and methylprednisolone group compared to the IVIG‐only and methylprednisolone‐only groups, before and after propensity score analysis (*p* < 0.05).

In contrast, another cohort (Cai et al. 2022) showed at the 1‐year follow‐up 100% of the methylprednisolone‐only group, 81.8% of the IVIG‐only group, and 91.1% of the IVIG and methylprednisolone combination group had mRS scores between 0 and 2. This shows a higher proportion of short‐term improvement in the methylprednisolone group, but the sizes of this group and the IVIG‐only group are much smaller than the combination therapy group (11, 11, and 56, respectively).

Several cohort studies (Dai et al. 2019; Huang et al. 2016; Huang et al. 2022; Lee et al. 2021; Pradhan et al. 2019; Titulaer et al. 2013; Zhang et al. 2018; Ariño et al. 2016; Hang et al. 2020; Huang et al. 2015; Kong et al. 2019; Lee et al. 2022; Melamud et al. 2020; Shin et al. 2013) consisted of cohorts of patients who were treated by multiple modalities of immunotherapy and no separate analysis for outcomes related to specific treatments. The proportion of patients treated with IVIG ranged from 41.7% to 96.2%. Overall, all of these studies demonstrated that either a majority of patients improved neurologically and cognitively over a long‐term period or demonstrated the average scores of patients improved in the long term, either by mRS score, ACE score, or symptomatic scores. In the study with the largest sample size of 577, 92% of the patients had IVIG included in their immunotherapy regime and 81% of the patients achieved mRS scores between 0 and 2 at the 2‐year follow‐up.

### 3.5 Seizure Incidence

The randomized controlled trial (Dubey et al. 2020) included 17 participants, of which 8 were randomly assigned to receive IVIG, and 9 were assigned to placebo. In the IVIG group, 75% of the patients achieved a reduction of seizure frequency greater than or equal to fifty percent compared to the placebo group, where 22% of the patients experienced a reduction of seizure frequency greater than or equal to 50% (*p* = 0.044, odds ratio = 10.5, 95% CI 1.1 to 98.9).

This is mirrored in a retrospective cohort of 56 participants (Wang et al. 2021), which found that the odds of patients treated with immunoglobulins achieving seizure‐free status after immunotherapy withdrawal were 5.852 times greater than those not treated with immunoglobulins (*p* = 0.027, odds ratio = 5.852, 95% CI 1.224 to 27.983). In another trial (Wong et al. 2010) all 9 participants had plasma exchange, IVIG and methylprednisolone treatment. All 8/8 patients with seizures experienced remission within a week of treatment.

Two more cohort studies (Zhu et al. 2020; Zhu et al. 2020) reported that their patient cohorts experienced fewer seizures after immunotherapy regimes including IVIG, but there was no further numerical information provided.

### 3.6 IVIG and Additional Treatments

As mentioned above in both the short‐term and long‐term outcomes, a high‐quality cohort (Gong et al. 2022) and a lower‐quality cohort (Cai et al. 2022) demonstrated that better outcomes were associated with the combined treatment of IVIG and corticosteroids. These findings were significant in the high‐quality paper. However, these studies had differing results on ICU admissions. The former study reports the highest percentage of patients admitted to ICU belonging to the combined therapy group, but these findings were statistically insignificant even after propensity score matching (*p* = 0.34). The latter reported the lowest percentage of ICU admissions in the group treated with combined therapy but there was no statistical analysis and the group sizes varied greatly.

In contrast to these studies, one cohort study (Wang et al. 2021), which looked at the patients’ mRS scores on admission and discharge, associated the addition of corticosteroids with poorer outcomes. The scores were equal for both groups at admission (median 4, range 3–5). At discharge, the IVIG monotherapy group improved overall with a median score of 2 (range 1–3), and the IVIG + methylprednisolone group did not improve with a median score of 4 (range 2–5). The difference was statistically significant (*p* < 0.001).

A prospective single‐arm trial (Lee et al. 2022) where all participants were treated with intravenous immunoglobulin for 5 days showed that on day 8, the mean mRS was 2.78 compared to a baseline mean of 3.44 (*p* = 0.004). However, by the day 29 follow‐up, out of 18 participants, 12 had required rescue therapy after 7 days due to either neurological deterioration or no improvement from baseline, demonstrating that two‐thirds of the patients benefitted from additional therapies. These therapies included corticosteroids, rituximab, and tocilizumab in various combinations.

Another nonrandomized trial (Zhang et al. 2019) demonstrated that patients who had therapeutic plasma exchange added to their immunotherapy regime of IVIG plus corticosteroids exhibited significantly better outcomes in the first two months following treatment (*p* < 0.05), although after 6 months there were no longer significant differences between treatment groups. To further add to evidence that therapeutic plasma exchange may be beneficial alongside IVIG, a nonrandomized trial (Wong et al. 2010) where all 9 participants had plasma exchange added to IVIG and methylprednisolone, all 8/8 patients experiencing seizures experienced remission, 3/3 reported hyponatremia cases resolved and 4/5 patients tested by ACE demonstrated improvement. Additionally, a cohort of 9 participants each described patients who were treated with corticosteroids, IVIG, and therapeutic plasma exchange. Out of 6 patients followed up, 3 showed substantial improvement based on neurological symptoms (Pham et al. 2011).

As stated previously, multiple studies measured the overall outcomes of cohorts treated with various immunotherapies, making it difficult to extract results regarding the impact of IVIG on additional therapies.

### 3.7 Adverse Events

Seven studies looked at adverse events related to the therapies used. Of these, three studies (Wong et al. 2010; Lee et al. 2021; Lee et al. 2022) did not note any adverse events concerning IVIG.

The RCT (Dubey et al. 2020) reported 1 mild to moderate headache attributed to IVIG infusion, while a nonrandomized trial (Lee et al. 2022) with over twice the number of participants receiving IVIG than the RCT, reported 5 adverse events that could be possibly or definitely related to IVIG infusions. This included 2 events of shivering during the infusion, 2 events of chest discomfort and one event of diplopia. All of these adverse events were transient and mild.

Gong et al. (2022) reported a total of 112 out of 374 patients treated by IVIG. One patient in the IVIG group had a deep venous thrombosis (DVT). All other adverse events were reported to be mild and did not affect treatment. These adverse events included infusion pain, skin rash and peripheral edema. Another cohort study (Cai et al. 2022) of 78 patients similarly described 4 adverse effects in the IVIG and methylprednisolone combined group and 1 adverse effect in the methylprednisolone group, but none in the IVIG‐only group—respective group sizes 56, 11 and 11.

In the cohort study by Wang et al. (2021) of 278 anti‐NMDAR encephalitis patients, 39% of patients treated with IVIG monotherapy experienced one or more episodes of clinical infection while in hospital compared to almost 64.4% in patients who received a combination of IVIG and methylprednisolone. The respective group sizes were 33 participants and 103 participants. Infection can be classed as an adverse effect, and this is significantly higher in the combined therapy group (*p* < 0.0001). The authors also report a significantly higher (*p* = 0.018) rate of noninfectious complications in the combined therapy group versus the IVIG group—76.25% and 61.90% respectively. Noninfectious complications are as listed: DVT/PE, gastric ulcer, electrolyte disorders, abnormal liver or kidney function, respiratory failure, hypoalbuminemia, and “others” which have not been specified.

### 3.8 Mortality Rates, Discharge Rates, and Antibody Types

Data on the impact of IVIG on discharge rates was not available in the included papers, and there were insufficient data to analyze the impact of IVIG on mortality rates. The antibody types of the patients were mentioned in all of the papers, but most papers did not provide comparative data between groups of patients of different antibody types. The randomized trial (Dubey et al. 2020) included patients of two antibody groups—anti LGI1 and anti‐CASPR2—but unfortunately all 3 patients with the latter antibody type were randomized to receive placebo so there are no comparisons to be made. Overall, there was not sufficient information to discuss the impacts of IVIG therapy on each antibody type.

## 4 Discussion

This review presents an analysis of the impacts of using IVIG in autoimmune encephalitis. IVIG, among other immunotherapy treatments, is often used when clinicians suspect autoimmune encephalitis, although the evidence to guide this use has not always been clear. In this condition with high morbidity and life‐long consequences for those affected, the available evidence has been synthesized to the above results with the hope to provide further clarity on the use of this treatment.

Study designs varied greatly, making it difficult to compare directly between studies, but some important conclusions can be drawn. Many of the research questions in this review were difficult to answer comprehensively with the quality of the evidence available, but the synthesized information shows us the following.

The available evidence indicates that IVIG is associated with improved neurological outcomes, both in the short term and long term, although there was only the small RCT by Dubey et al. (2020) and the paper by Gong et al. (2022) were deemed to be high quality; inferences made from other cohort studies must be considered carefully.

Two papers, including one RCT, provide evidence that the use of IVIG in patients with autoimmune encephalitis can help to reduce seizures. The quality of the randomized trial is high, and there was statistical significance in the reduction of seizures, but the authors were unable to reach a sufficient sample size of 30 based on 89% power, and instead recruited a total of 17 participants. Of the studies that considered adverse events, the adverse events associated with IVIG were mostly mild, transient, and did not result in any treatment changes. There was only one serious adverse effect reported overall: a DVT. This seems reflective of the literature, such as this 10‐year retrospective cohort (Palabrica et al. 2013), demonstrating that largely IVIG may be safe for use in this condition.

Considering that two cohort studies (Cai et al. 2022; Wang et al. 2021) indicated higher rates of adverse effects in patients treated by corticosteroids compared to IVIG monotherapy, it may be true that IVIG is safer for certain patients than corticosteroids. Neither of these papers was considered high‐quality, but the risks of corticosteroid treatment are well‐known (Poetker and Reh 2010) and therefore highlight the need for further research to evaluate whether IVIG could be a safer option for certain patients.

Regarding the impact of IVIG on additional therapies there is conflicting evidence that adding corticosteroid therapy to IVIG is associated with significantly better neurological outcomes than those of IVIG therapy alone, but the evidence from a high‐quality cohort (Gong et al. 2022) supports this combined therapy. Similarly, there is evidence suggesting that IVIG alone can be associated with improved outcomes in some patients compared to placebo. However, in other patients, additional therapies such as corticosteroids, rituximab, and tocilizumab may provide further improvement. The addition of therapeutic plasma exchange to IVIG and corticosteroids may be beneficial.

Not all authors chose to supply treatment outcomes relating to each immunotherapy type. This makes it difficult to find associations between treatments and outcomes. However, even when authors do include individual immunotherapy outcomes, it is imperative to understand that there are factors that result in the selection of specific immunotherapy treatment for patients, such as the clinical severity of the patient's disease, or the preference of the prescribing clinician. Therefore, there are many confounders when examining associations between IVIG and neurological outcomes.

Additionally, most authors did not provide detail on the timing of the administration of IVIG or other therapies, or the dosage of the treatment, making the results far less comparable than desired.

A meta‐analysis performed by Nosadini et al. (2021) has also demonstrated that the evidence supporting the use of immunotherapy in AE exists, but similarly confirmed the data is lacking regarding the efficacy of specific immunotherapies or certain combinations of immunotherapies. The meta‐analysis demonstrates that immunotherapy as a whole is effective, while this review has illustrated there are links specifically between the use of IVIG and an improvement in neurological outcomes.

### 4.1 Limitations

A major limitation of this review is the design, quality, and size of the included studies. In particular, most of the studies are observational and only one study was a randomized controlled trial. The likely reason for this is that as autoimmune encephalitis is a relatively uncommon and acute condition, recruiting for trials is difficult. Additionally, due to the heterogeneity of the study designs, the comparisons that can be made between papers are extremely limited and must be made cautiously.

Linking in closely to this, there is very little comparison that can be made regarding the response to IVIG of different antibody subtypes of encephalitis. While this was initially one of the aims of this review, it has quickly become apparent that while various different antibody subtypes were mentioned in the papers included, there were very few comparisons that could be reliably made between such heterogeneous papers.

There is also a lack of information available regarding important clinical outcomes, such as time to discharge and mortality rates. These outcome measures would be useful to evaluate treatment efficacy as they would provide a comparable benchmark between treatment regimes.

Another limitation to note is that the mRS scoring system, which many of the studies adopted, was validated in the context of strokes, and therefore may not be accurate to reflect outcomes of encephalitis patients.

Moving forwards, there is a need for research of a higher quality. Ideally, questions related to treatment outcomes will be answered by well‐designed randomized controlled trials. Additionally, high quality observational studies can provide useful information. For example, the paper written by Gong et al. (2022) was deemed to be high quality due to the actions taken to match the baselines of the two groups and carrying out propensity score matching to control for potential confounders.

For further scoping of the research in the future, it may be appropriate to narrow down further to studies who have reported individual patient treatment data as multiple papers included in this review reported patient outcomes simultaneously for groups of patients who received various combinations of treatment, reducing the ability to analyze treatment outcomes.

## 5 Conclusion

This systematic review evaluated the currently available evidence regarding the use of intravenous immunoglobulin in adults who have autoimmune encephalitis. The designs of these studies varied, limiting comparability. However, the available evidence demonstrates that IVIG is associated with improvements in neurological outcomes of patients with autoimmune encephalitis and could be more effective when used in conjunction with corticosteroids and other immunotherapies. The available evidence also demonstrates that although IVIG can have serious adverse effects, they seem to be few and mostly mild.

This review demonstrates that there is a continued lack of clarity surrounding the efficacy of IVIG. Therefore, there is serious need for further clinical trials and high‐quality prospective studies to draw stronger conclusions about whether intravenous immunoglobulins should be used to treat adults with autoimmune encephalitis.

## Author Contributions

**Anahat Kalra**: conceptualization, data curation, writing–original draft, methodology, investigation. **Olivia Mackay**: methodology. **Emma Thomas‐Jones**: supervision, project administration, writing–review and editing, resources, conceptualization. **Tom Solomon**: writing–review and editing. **Paula Foscarini‐Craggs**: investigation, supervision, project administration, writing–review and editing, resources, conceptualization.

## Conflicts of Interest

The authors of this review declare no conflict of interest.

### Peer Review

The peer review history for this article is available at https://publons.com/publon/10.1002/brb3.70491

## Supporting information



Supporting Information

## Data Availability

The data that supports the findings of this study are available in the Supplementary Material of this article
